# Visual short-term memory binding deficit with age-related hearing loss in cognitively normal older adults

**DOI:** 10.1038/s41598-019-49023-1

**Published:** 2019-08-29

**Authors:** David G. Loughrey, Mario A. Parra, Brian A. Lawlor

**Affiliations:** 1 Global Brain Health Institute, Trinity College Dublin, Dublin, Ireland/University of California, San Francisco, USA; 2 Trinity College Institute of Neuroscience, Trinity College Dublin, Dublin, Ireland; 3 School of Psychological Sciences and Health, University of Strathclyde, Glasgow, UK; 4 Programa de Psicología, Universidad Autónoma del Caribe, Barranquilla, Colombia; 5 School of Medicine, Trinity College Dublin, Dublin, Ireland; 6 Mercer’s Institute for Successful Ageing, St James Hospital, Dublin, Ireland

**Keywords:** Cognitive ageing, Predictive markers

## Abstract

Age-related hearing loss (ARHL) has been posited as a possible modifiable risk factor for neurocognitive impairment and dementia. Measures sensitive to early neurocognitive changes associated with ARHL would help to elucidate the mechanisms underpinning this relationship. We hypothesized that ARHL might be associated with decline in visual short-term memory binding (VSTMB), a potential biomarker for preclinical dementia due to Alzheimer’s disease (AD). We examined differences in accuracy between older adults with hearing loss and a control group on the VSTMB task from a single feature (shapes) condition to a feature binding (shapes-colors) condition. Hearing loss was associated with a weaker capacity to process bound features which appeared to be accounted for by a weaker sensitivity for change detection (A’). Our findings give insight into the neural mechanisms underpinning neurocognitive decline with ARHL and its temporal sequence.

## Introduction

Age-related hearing loss (ARHL), the third most common chronic health condition among older adults^1^, has been recognized as a potential risk factor for dementia^1–3^. Effective management of ARHL could potentially have the biggest public health impact for any modifiable dementia risk factor^3^. However, it is not clear how ARHL is associated with dementia and there are several different hypotheses^2^. Measures sensitive to early neurocognitive changes associated with ARHL that identify risk of neurocognitive impairment would help to elucidate the mechanisms underpinning this relationship and would be valuable diagnostically and in clinical trials.

A challenge in developing accurate biomarkers of dementia risk is that executive neurocognitive networks may provide compensatory mechanisms which mask or delay clinical expression of neuropathological-related lesions^4^. Epidemiological and experimental evidence suggests that such networks are relatively maintained with ARHL because they are increasingly recruited for auditory processing to the detriment of lower-level processes such as encoding in working memory which are disrupted^5^. This may lead to an underestimation of cognitive decline following ARHL in its earlier stages and of the effectiveness of intervention when relying on traditional neuropsychological instruments to assess outcomes^6^.

Visual Short-Term Memory Binding (VSTMB) is a function responsible for binding features of an object temporarily in working memory^7^ and is sensitive to Alzheimer’s disease (AD) along its continuum beginning with the pre-hippocampal stages^8,9^. Encoding of bound visual features occurs automatically without reliance on executive resources^10^ but can be disrupted by increased cognitive load^11^ including that due to processing verbal stimuli^12^ as occurs in ARHL. Hence, the VSTMB test may unveil the neurocognitive impact of ARHL and more reliably identify risk of dementia due to AD during preclinical stages. In this study, we hypothesized that ARHL is associated with weaker VSTMB in cognitively normal adults.

## Methods

### Participants

Volunteers in this study were community-dwelling adults over the age of 50 recruited from the general population through community organizations and audiometric clinics for a study on ARHL and cognition. There were 25 participants in the hearing loss group (HLG) and 18 in the control group (CG). Volunteers were excluded from the study if they had a history of brain injury, epilepsy, stroke, neurological conditions, a history of drug/alcohol abuse, hospitalization for mental/emotional problems in the previous five years, if they were taking certain medications for a psychiatric condition, if they had possible cognitive impairment (based on a global cognitive z-score of <−1.5 SD on the neuropsychological assessment tests) or if they had a congenital/pre-lingual hearing loss or loss due to injury or disease. The Faculty of Health Sciences Research Ethics Committee of Trinity College Dublin approved all study protocols. The study was conducted in accordance with the 1964 Declaration of Helsinki, and its later amendments. Written informed consent was obtained from all participants. Testing with the VSTMB task took place between October 2016 and January 2017.

### Background assessment

Demographic data collected included age, sex, and education (both years and highest attainment). Self-rated measures were included of physical and mental health, alcohol consumption and smoking. *Sleep quality* was assessed using the Pittsburgh Sleep Quality Index (PSQI)^S1^; *pre-morbid IQ* using the National Adult Reading Test (NART)^S2^; *frailty* with the Survey of Health, Ageing and Retirement in Europe (SHARE) Frailty Instrument^S3^; *depression* with the 10 item Center for Epidemiologic Studies Depression Scale (CESD-10)^S4^; *anxiety* using the Hospital Anxiety and Depression Scale-Anxiety subscale (HADS-A)^S5^; *apathy* with the Apathy Evaluation Scale – Self-rated (AES-S)^S6^; *social network* with the Lubben Social Network Scale (LSNS)^S7^; *loneliness* with the 6-item De Jong Gierveld Loneliness Scale (DJGLS)^S8^; *boredom proneness* using a self-report question with a four-point scale^S9^; *perceived stress* with the Perceived Stress Scale-4 item (PSS-4)^S10^. The Hearing Handicap Inventory for the Elderly Screening Version (HHIE-S) assessed self-reported hearing loss^S11^.

### Audiometric assessment

Pure-tone audiometry was used to assess peripheral ear function. The assessment was conducted by audiologists and followed the standards of the British Society of Audiology and of the American National Standards Institute. Participants’ ears were checked by otoscope. Pure-tone air conduction decibel thresholds were obtained in each ear at frequencies 0.5, 1, 2, 3, 4, 6, and 8 kilohertz with calibrated audiometers (Grayson Sadler GSI 61 or Interacoustics Callisto) and TDH 39 supra-aural earphones (Telephonics, Huntington, New York). The World Health Organization (WHO) criteria for hearing loss were used: pure-tone average (PTA) ≥ 26 dB for 0.5, 1, 2 & 4 kHz in the better ear^13^. Participants meeting these criteria were allocated to HLG and those below this threshold were allocated to CG. We also calculated the PTA of these frequencies for the worse ear. The PTA for low (0.25, 0.5 & 1 kHz) and high frequencies (3, 4, & 6 kHz) for both ears were included to provide an estimate of low and high frequency loss.

### Neuropsychological assessment

We conducted a neuropsychological assessment of the main cognitive domains. *General cognitive function* was assessed using the Montreal Cognitive Assessment (MoCA)^S12^ and a composite z-score was calculated from tests of the following domains: *episodic memory* was assessed using the Free and Cued Selective Reminding Test (FCSRT)^S13^ with immediate and delayed recall (after 30 minutes) subsets and Wechsler Memory Scale-III (WMS-III) spatial span forward subset^S14^; e*xecutive function* was assessed using the Visual Reasoning subtest of the Cambridge Mental Disorders of the Elderly Examination (CAMDEX) battery^S15^, the Sustained Attention to Response task (SART)^S16^, the phonological fluency test from the MoCA^e12^ and the WMS-III spatial span backward subset^S14^; *processing speed* was assessed using a computer-based choice-reaction time test (CRT) which included motor and cognitive components^S17^ and mean response time (RT) from the SART^S16^; *language* was assessed using the Boston Naming Test 60-item version^S18^ and the semantic (animals) fluency^S19^ and *visuospatial ability* was assessed using the Medical College of Georgia (MCG) Complex Figure test (copy only)^S20^. None of the tests used auditory stimuli except the MoCA (we used scores both including and excluding audiological items)^S21^.

### VSTMB test

**Figure 1:**
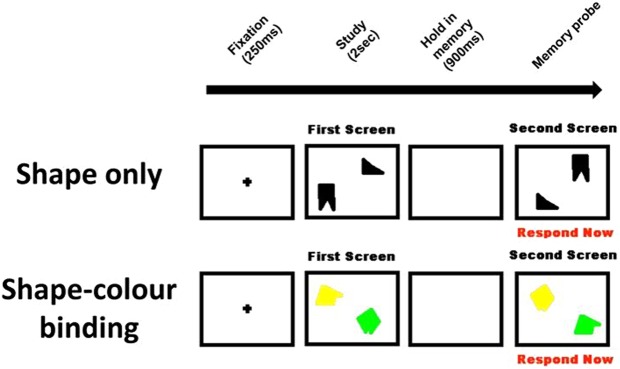
The two conditions (shapes and shapes-colors binding) of the Visual Short-Term Memory Binding Task.

Using a computer, participants were administered a screening test (to ensure capacity to form bindings in perception) and the VSTMB test which was the same as that used by Parra *et al*. (2010)^9^. Participants were asked to remember two study visual arrays (2000 ms) and after a brief pause (900 ms) to detect if a change has occurred when visually prompted with a test array (Fig. 1). The first condition consisted of two shapes-only arrays. The second condition consisted of two colored shapes arrays. In both conditions, participants were instructed to state verbally whether or not the stimulus in the test display was the ‘same’ (as) or ‘different’ (from) the stimulus in the study display. Participants were allowed to respond in their own time. At the beginning of each trial, a fixation screen appeared for 250 ms. Changes in the test arrays consisted of new features replacing studied features (shape-only) or features swapping across items (shape-color binding). For the first condition, the two arrays were randomly selected from a set of eight six-sided random polygons shapes. For the second condition, the two arrays were selected from the same selection of shapes and from a set of eight colors. Both the shapes and binding conditions consisted of 15 practice trials followed by 32 test trials. Of these 32 trials, 16 were ‘same trials’ and 16 were ‘different trials.’ Stimuli were presented at 1° of visual angle and fell within an area of 10°. Participants were instructed to ignore the location of the stimulus on the screen which varied randomly across trials and between study and test displays. The test took approximately 16 minutes to complete.

### Statistical analysis

We compared background and neuropsychological data, using the unpaired t-test or the χ^2^ test. Normality was examined using the Kolmogorov-Smirnov test and by visual inspection of the Q-Q plots and the data distribution in the histograms. Non-normal data were either transformed or analyzed using non-parametric tests, as appropriate. All statistical analyses were conducted using the Statistical Package for Social Sciences version 22 (SPSS Inc., Chicago, IL, U.S.A.).

We used a linear mixed model to conduct the primary analysis to assess difference between groups across VSTMB conditions (shapes to binding). As fixed effects in the model, we entered condition, group and a condition by group interaction term. Subject was entered as a random effect. Age, sex and years of education were entered as covariates. Residual plots were inspected for deviations from homoscedasticity or normality. We constructed another model with the slope added as a random factor. Models were fitted and compared based on the −2 Restricted Log Likelihood and Akaike’s Information Criterion. The first model was deemed the better fit. We selected a diagonal structure as the covariance structure for the error terms based on the above criteria.

As a secondary analysis, we assessed the differences between groups on all VSTMB outcomes using ANCOVA with the same covariates. We conducted an additional analysis assessing sensitivity for change detection^9^ following Signal Detection Theory measures^14^. A’ was selected as the sensitivity measure^15^ and was calculated according to the formulas provided by Xu^16^ which do not have indeterminacy when a participant does not make false alarms. Poor performance accounted for by low sensitivity would suggest difficulties in keeping the signal separate from the noise in working memory^9^.

Using Pearson’s *r* or Spearman’s correlation coefficient, we explored associations between shapes and binding accuracy with hearing loss (WHO PTA for entire sample) along with age and other variables recognized as potential modifiable dementia risk factors (depression, level of education, physical inactivity, smoking, and social engagement)^3^. We explored associations between shapes and binding accuracy with outcomes on several tests recommended for AD assessment (FCSRT delayed free recall, phonemic/semantic fluency, BNT and MoCA)^4^ across groups^17^. We made adjustments for false discovery rates. We also compared VSTMB high and low HLG performers and the CG on background and neuropsychological data.

## Results

### Group characteristics

Groups were well matched on background factors (Table 1). A significant difference existed between groups on all audiological outcomes (P < 0.001). Seventeen (68%) of the participants in the HLG and none in the CG wore hearing aids. Thirteen (52%) participants in the HLG and thirteen (72%) in the CG reported having previously experienced tinnitus. No participants reported difficulty with vision. All participants passed the perceptual binding screening assessment. No significant difference was observed between groups on any traditional neuropsychological test except for visuospatial ability where the HLG performed more poorly (mean [SD], 24.22 [4.38] vs 27.06 [4.5]; P = 0.045) (Table 2).

**Table 1:** Background data for the two groups of participants.

	HLG *M (SD)*	CG *M (SD)*	*p*
**Demographic**			
N	25	18	—
Age	72.56 (5.79)	69.11 (6.63)	0.08
Sex (*female/male)*	14/11	14/4	0.25
Education *(years)*	13.58 (3.62)	14.44 (3.09)	0.42
Education *(level)*	2.8 (0.76)	2.94 (0.73)	0.45
**Audiological**			
WHO better ear PTA	49.9 (17.23)	13.61 (6.61)	**<0.001**
WHO worse ear PTA	63.2 (25.76)	19.17 (8.73)	**<0.001**
Low freq. better ear PTA	54.64 (13.29)	12.64 (9.38)	**<0.001**
Low freq. worse ear PTA	68.93 (18.62)	19.89 (17.47)	**<0.001**
High freq. better ear PTA	75.6 (14.8)	34.02 (16.56)	**<0.001**
High freq. worse ear PTA	90.12 (21.41)	42.87 (19.36)	**<0.001**
Self-rated hearing *(HHIE-S)*	20.0 (8.43)	4.44 (6.49)	**<0.001**
**Health and psychosocial function**			
Self-rated physical health	3.52 (1.01)	3.83 (1.04)	0.34
Self-rated mental health	3.96 (0.84)	3.94 (1.11)	0.89
Physical inactivity level	2.04 (1.02)	2 (0.77)	0.97
Alcohol consumption *(yes/no)*	19/6	14/4	>0.99
Alcohol units *(per wk)*	8.49 (7.11)	12.64 (11.74)	0.26
Smoker current *(yes/no)*	1/24	0/18	>0.99
Smoker former *(yes/no)*	10/15	7/11	>0.99
Sleep quality *(PSQI)*	5.24 (3.02)	4.78 (2.53)	0.66
Pre-morbid IQ *(NART)*	112.87 (6.62)	115.17 (5.38)	0.23
Self-rated memory	3.32 (0.85)	3.56 (0.86)	0.35
Frailty *(SHARE score)*	0.26 (0.87)	0.21 (1.07)	0.69
Depression *(CESD-10)*	4.24 (3.02)	4.83 (4.46)	0.95
Anxiety *(HADS-A)*	3.48 (2.58)	3.83 (3.5)	0.96
Apathy *(AES-S)*	26.92 (4.65)	27.83 (7.21)	0.9
Social network *(LSNS)*	20.56 (5.55)	19.5 (6.17)	0.9
Loneliness *(DJGLS)*	0.32 (0.69)	0.83 (1.62)	0.39
Boredom proneness *(Conroy)*	1.36 (0.57)	1.61 (0.7)	0.21
Perceived stress *(PSS-4)*	3.08 (2.18)	2.33 (2.72)	0.14

Means (M) and standard deviations (SD) for the control group (CG) and the hearing loss group (HLG) on background data. AES-S, Apathy Evaluation Scale – Self-rated; CESD-10, Center for Epidemiologic Studies Depression Scale – 10 item; Conroy, Conroy Boredom proneness; DJGLS, 6-item De Jong Gierveld Loneliness Scale; HADS-A, Hospital Anxiety and Depression Scale-Anxiety subscale; HHIE-S, Hearing Handicap Inventory for the Elderly Screening Version; LSNS, Lubben Social Network Scale; NART, National Adult Reading Test; PSQI, Pittsburgh Sleep Quality Index; PSS-4, Perceived Stress Scale-4 item; PTA, Pure-tone average; SHARE, Survey of Health, Ageing and Retirement in Europe Frailty Instrument; WHO, World Health Organisation.

**Table 2:** Neuropsychological data for the two groups of participants.

	HLG *M (SD)*	CG *M (SD)*	*p*
**Current sleepiness**			
Stanford Sleepiness Scale	1.72 (0.89)	1.89 (0.9)	0.48
**Episodic memory**			
FCSRT immediate free recall	33.28 (7.19)	34.39 (4.35)	0.53
FCSRT immediate total recall	47.64 (1.41)	48 (0)	0.13
FCSRT delayed free recall	12.52 (2.74)	12.11 (2.06)	0.6
FCSRT delayed total recall	15.92 (0.4)	16 (0)	0.4
WMS-III spatial span forward	7.08 (2.04)	7 (1.82)	0.9
*Composite z-score*	0.004 (0.85)	−0.01 (0.58)	0.97
**Executive function**			
CAMDEX VR	3.68 (1.15)	3.83 (1.25)	0.68
SART commission errors	3.12 (2.37)	3.89 (2.97)	0.47
SART omission errors	6.24 (5.61)	10.33 (10.34)	0.25
SART total errors	9.36 (7.4)	14.22 (11.56)	0.18
Phon. fluency *(MoCA)*	15.04 (4.79)	14.22 (4.17)	0.56
WMS-III SS backward	6.44 (1.76)	6.67 (1.82)	0.68
WMS-III SS total	13.52 (3.33)	13.67 (3.2)	0.89
*Composite z-score*	0.06 (0.59)	−0.08 (0.7)	0.47
**Processing speed**			
CRT motor MRT (*ms*)	302.97 (76.65)	297.30 (56.19)	0.91
CRT cognitive MRT (*ms*)	485.57 (66.29)	501.85 (66.15)	0.36
CRT total MRT (*ms*)	788.46 (85.07)	797.43 (84.53)	0.74
SART MRT (*ms*)	334.13 (82.29)	319.14 (62.53)	0.6
**Language**			
BNT	55.64 (3.6)	56.5 (2.33)	0.59
Semantic fluency *(animals)*	22.84 (5.45)	22.83 (6.36)	>0.99
*Composite z-score*	−0.06 (0.87)	0.08 (0.69)	0.59
**Visuospatial ability**			
MCG complex figure copy	24.22 (4.38)	27.06 (4.5)	**0.045**
**Global cognition**			
MoCA	25.96 (2.85)	26 (2.74)	0.96
MoCA adj.	17.72 (1.79)	17.72 (2.02)	>0.99
*Composite global z-score* ^+^	−0.04 (0.53)	0.06 (0.42)	0.51

Means (M) and standard deviation (SD) for the control group (CG) and the hearing loss group (HLG) on the neuropsychological data. ^+^Composite global z-score calculated from the mean of the composite scores for episodic memory (except FCSRT total scores), executive functions (except SART and WMS-III spatial span total scores) and language, and from processing speed (CRT total MRT), and visuospatial ability. BNT, Boston Naming Test 60-item version; CAMDEX VR, Cambridge Mental Disorders of the Elderly Examination battery Visual Reasoning subtest; CRT, choice-reaction time test which included motor, cognitive and total mean reaction times (MRT) in milliseconds (ms); FCSRT, Free and Cued Selective Reminding Test with free and total (cued) recall scores; MCG, Medical College of Georgia Complex Figure copy test; MoCA, Montreal cognitive Assessment with an adjusted score (audiological items removed); SART, Sustained Attention to Response Task commission, omission and total error scores and mean reaction time (MRT) in milliseconds (ms); WMS-III, Wechsler Memory Scale-III spatial span forward, backward and total scores.

### VSTMB results

Prior to adding the interaction term, there was no significant effect for any variable except condition (Table 3). When the interaction term was added to the model, it was the only significant variable, with HLG demonstrating a greater drop in accuracy from the shapes to the binding condition (β = −0.064, 95% CI = −0.125 to −0.003; P = 0.04).

**Table 3:** VSTMB task outcomes for the two groups of participants.

Linear mixed effects model of change in accuracy from shapes to binding conditions						
	*β*	*95% CI*	Significance test			
			*t*		*p*	
Condition	−0.060	−0.091 to −0.029	−3.86		**<0.001**	
Group	−0.018	−0.048 to 0.011	−1.26		0.22	
Age	0.000	−0.003 to 0.002	−0.19		0.85	
Sex	−0.017	−0.047 to 0.014	−1.11		0.28	
Education (*years*)	0.001	−0.003 to 0.006	0.55		0.59	
	***β***	***95% CI***	***t***		***p***	
Condition	0.041	−0.060 to 0.142	0.82		0.42	
Group	0.057	−0.021 to 0.135	1.48		0.15	
Group*Condition	−0.064	−0.125 to −0.003	−2.11		**0.04**	
Age	−0.0002	−0.003 to 0.002	−0.19		0.85	
Sex	−0.017	−0.047 to 0.014	−1.11		0.28	
Education (*years*)	0.001	−0.003 to 0.006	0.55		0.59	
ANCOVA analysis of difference on each VSTMB outcome						
	**HLG**		**CG**		**Significance test**	
	***M (SD)***	***Range***	***M (SD)***	***Range***	***F***	***p***
Shape MRT (*ms*)	2153.96 (427.43)	1561–3514	2061.61 (319.21)	1507–2792	0.26	0.61
Shape Acc.	0.95 (0.05)	0.78–1	0.96 (0.04)	0.88–1	0.24	0.63
Shape *A’*	0.97 (0.05)	0.77–1	0.98 (0.03)	0.88–1	0.74*	0.39
Bind MRT (*ms*)	2562.36 (550.03)	1832–4455	2330.11 (559.68)	1435–3475	0.78	0.38
Bind Acc.	0.86 (0.11)	0.62–1	0.93 (0.06)	0.78–1	4.92	**0.03**
Bind *A’*	0.8 (0.23)	0.23–1	0.92 (0.08)	0.7–1	3.66	0.06

The visual short-term memory binding (VSTMB) task outcomes for the control group (CG) and the hearing loss group (HLG). Linear mixed models were used to examine the primary outcome of change in accuracy from shapes to binding conditions between groups. The ANCOVA models were used to assess all the outcomes of the VSTMB test as a secondary analysis. Age, sex and years of education were included in linear mixed and ANCOVA models as covariates. Mean Reaction Time (MRT) for shapes transformed to inverse of square root to account for non-normality. Binding *A’* data transformed to a squared scale. *Assessed using rank analysis of covariance.

Results of the secondary (ANCOVA) analyses for each VSTMB outcome (Table 3) showed no significant difference between groups on the shapes-only condition outcomes. For the binding condition, we found no significant difference in reaction time. The HLG demonstrated poorer performance compared to CG on binding accuracy (0.86 [0.11] vs 0.93 [0.06]; P = 0.03). We found no significant difference for the sensitivity measure (A’) on shapes-only condition; however, a lower sensitivity for the HLG approached significance on the binding condition (0.8 [0.23] vs 0.92 [0.08]; P = 0.06).

### VSTMB associations with dementia risk factors and assessment tools

Compared to age and other, modifiable, dementia risk factors only hearing loss was associated with binding accuracy whereas only social engagement was significantly associated with shapes accuracy. (Supplementary Table S1). When compared with other AD assessment tools, only phonemic fluency was significantly correlated with binding accuracy in the HLG (Supplementary Table S2). These findings remained after removal of low performers from the CG. None of the above associations remained significant after adjustment for false discovery rate^17^. We included correlations between shapes/binding accuracy and all background and neuropsychological variables in Supplementary Tables S3 and S4.

### High vs low VSTMB performers

The total sample mean (0.89) was used as the cut-off point in binding accuracy in the HLG which gave 11 HLG-high and 14 HLG-low performers (Supplementary Table S5 and S6). Outcomes for the three groups on background measures were the same (P > 0.10) except NART scores which trended toward significance (HLG-low = 110.88 [6.25], HLG-high = 115.39 [6.48], CG = 115.17 [5.38]; P = 0.09). For neuropsychological tests, outcomes were the same across groups (P > 0.10) with the exceptions of phonemic fluency (HLG-low = 13.29 [4.2], HLG-high = 17.27 [4.74], CG = 14.22 [4.17]; P = 0.07) and the MCG complex figure copy task (HLG-low = 23.5 [4.26], HLG-high = 25.14 [4.57], CG = 27.06 [4.5]; P = 0.09) which also trended toward significance. These findings remained unchanged with removal of low performers from the CG (N = 4). When only HLG-high performers and HLG-low performers were compared, there were no differences (P > 0.10) with the exceptions that the HLG-low had greater low-frequency hearing loss (both P < 0.10) and poorer NART scores (P = 0.09) and phonemic fluency (P = 0.04).

## Discussion

Compared to controls, the HLG showed poorer capacity to process bound features in visual short-term memory. We found no difference in accuracy between groups on the shapes-only condition. The two groups were otherwise matched for background characteristics and neuropsychological performance (with the exception of the MCG complex figure copy task). All participants passed the perceptual binding screening assessment. Therefore, decline in processing bound features was more likely due to a weaker capacity to maintain a strong signal-to-noise ratio in working memory than to perceptual difficulties. This pattern has been observed previously only in asymptomatic carriers of the E280A single *presenilin-1* mutation which leads in 100% of cases to autosomic dominant familial AD^9^. In this study, Parra and colleagues^9^ also reported poorer (but not significantly poorer) performance for the asymptomatic carriers compared to controls on an identical complex figure copy task. AD and stroke studies indicate that performance on drawing tasks is modulated by several frontal and temporal-parietal cortex regions including the right temporal and parahippocampal gyri^18–22^ in which atrophy has been observed with ARHL^23,24^.

A meta-analysis of epidemiological studies reported that ARHL was associated with decline in multiple domains of cognition including working memory and visuospatial ability^2^. However, there is limited research into what initial changes may occur in neurocognitive function with ARHL prior to a stage where decline may be observed in multiple domains of cognition. The results of this study suggests that altered VSTMB may be a feature of such early changes in neurocognitive function with ARHL. Our findings are consistent with previous research. It is known that in ARHL the brain undergoes functional reorganization and that this might negatively impact on the ability to retain information in memory (i.e. maladaptive plasticity)^23,25–27^. A small number of neuro-imaging studies have reported atrophy in neural regions that are important for memory with ARHL^23,24,26,27^.

Two studies that examined data from the Baltimore Longitudinal Study of Aging reported a faster decline in the temporal lobes in regions that are critical for memory^23,26^. One of these studies reported that ARHL was associated with accelerated atrophy (comparable to those developing mild cognitive impairment) in the parahippocampal gyrus^23^ which is part of the ventral stream and contributes to the encoding and maintenance of bound information in working memory^28–30^. The other study reported that poorer midlife hearing was associated with atrophy in the right hippocampus and in the entorhinal cortex^26^. Another recent study using data from the Alzheimer’s Disease Neuroimaging Initiative (ADNI) database also reported that ARHL was associated with elevated cerebrospinal fluid tau levels and atrophy of the hippocampus and entorhinal cortex^27^. The entorhinal cortex is affected in the early stages of AD^31^ but cortical thickness of this region has also been linked with memory scores independent of the level of β-amyloidosis and tauopathy^32^.

A limited number of studies have been conducted examining the link between neural changes with ARHL and changes in cognitive function in humans^33–35^. One such study reported a correlation of poorer function in several cognitive domains including episodic memory and visuoconstructive ability with atrophy in the cingulate cortex^35^, a neural region important for maintenance in working memory^36,37^. Support for a causal relationship between ARHL and neurocognitive decline comes from several mouse studies which report brain atrophy, impaired neurogenesis (including in the hippocampus) and increased expression of phosphorylated tau following hearing loss along with impaired learning and memory^38–42^. If we consider that VSTMB relies on a network which involves regions known to be functionally disrupted in ARHL individuals and in prodromal AD^7^, then the selective VSTMB deficits observed in this study may be indexing such a negative functional reorganization which is thought to be a potential mechanism linking ARHL to dementia. Such a hypothesis will need investigation.

Multiple hypotheses exist as to how ARHL and dementia may be connected. There may be a common causal mechanism such as vascular determinants, a mechanistic pathway such as neural reorganization due to hearing loss or a mediating factor such as social isolation following ARHL^6^. Neuro-imaging evidence suggests that this functional reorganization may be driven by an impoverished auditory input or by the attentional load associated with difficulties in perceiving speech following ARHL^25,43^. Findings from our exploratory analyses are consistent with this. Those in the HLG who performed poorly on the VSTMB task had greater hearing loss in the lower frequencies (crucial for speech) indicating further advancement in the ARHL pathophysiological process. Additionally, they had lower phonemic fluency scores, possibly reflecting the decline in phonological abilities previously observed in ARHL^5^.

Higher cognitive load in auditory working memory when processing speech may draw resources from ventral stream regions^44^ which maintain feature binding^45^. Also, altered visual attention to assist speech perception following early stage ARHL may drive cross-modal reorganization along the ventral visual stream in temporal regions associated with auditory processing^25,46^. Interestingly, mild AD patients present altered visual attention when processing bound (but not unbound) features, possibly reflecting inefficient cortical mechanisms responsible for encoding bindings^47^.

Alternatively, a common pathophysiological mechanism may affect both the inner ear and neural regions sub-serving feature binding. While the primary risk factor for both ARHL and AD dementia is age^48^, the VSTMB task has been demonstrated to be insensitive to ageing^49^. Additionally, pathophysiologic features of AD have been observed in central auditory neural regions but not in the peripheral auditory structures^50^. Genetic risk factors may account for such an association. For example, ApoE e4 (*apolipoprotein E-epsilon4*) is strongly linked in isoform-dependent manner with sporadic AD^51,52^ and ARHL^53,54^, possibly through changes in cholesterol homeostasis^55^ or hypercholesterolemia in the main vasculature and associated atherosclerosis^56,57^. Other possible common mechanisms include the metabotropic glutamate receptor gene which is linked to both ARHL and AD via the glutamatergic pathway or mitochondrial dysfunction via the SIRT3 pathway^48^.

### Limitations

The primary limitation of our study is small sample sizes and a small number of VSTMB trials which may have resulted in an underestimation of the difference between groups. Additionally, while we found a weaker capacity to form visual bindings with ARHL, we cannot deduce from these findings how ARHL and impaired VSTMB are connected. Our findings provide some support for the hypothesis that ARHL mechanistically affects cognitive function based on prior literature as reported here. Limited research has been conducted on changes in cognitive processing with ARHL prior to decline in performance on more general cognitive tests such as the MoCA as observed in epidemiological studies. Further research is warranted to examine if altered visual short-term memory processing is a feature of early cognitive decline following ARHL. Neuro-imaging studies examining the neural correlates of binding in an ARHL sample compared to controls and AD samples would be informative. Any differences or similarities in neural correlates of binding across ARHL and AD groups matched in behavioral performance would help to elucidate the underlying pathophysiological processes linking ARHL with dementia. Genetic markers for both ARHL and AD could also be assessed. Furthermore, longitudinal studies are required to assess the validity of impaired VSTMB in predicting future risk of dementia with ARHL.

The VSTMB test is purely visual making it appropriate for use with ARHL patients. In our sample, maintained executive resources could not compensate for weaker binding capacity. Also, the VSTMB test does not have any linguistic components meaning that it can be used globally and in developing countries which are preferentially affected by both ARHL and dementia. It is insensitive to normal cognitive ageing, education and cultural background^45^. Furthermore, VSTMB is not impaired in other age-related clinical conditions including depression, vascular dementia, dementia with Parkinson’s disease, dementia with Lewy bodies and frontal lobe dementia^45^.

Clinical trials aimed at maintaining or rehabilitating cognitive function in ARHL could include VSTMB as a target for therapeutic success or as a preclinical marker to identify potential participants. Hearing aids can reduce attentional costs, particularly when equipped with algorithms to improve speech-in-noise perception^5^. Also, benefits for visuospatial working memory have been noted^58^. However, the majority of the HLG reported wearing hearing aids suggesting that additional interventions may be required.

## Conclusions

In conclusion, we found a decline in VSTMB with hearing loss which has only previously been reported in AD samples. To the best of our knowledge this is the first study to link ARHL with a potential preclinical cognitive test for AD. Further research is warranted to examine the mechanism underpinning the relationship of ARHL with VSTMB and examine it as a potential biomarker for future dementia.

## Supplementary information

Supplementary Information

## Data Availability

Following publication, anonymized data will be shared by request from any qualified investigator.
