# Supplementary material for: Visual short-term memory binding deficit with age-related hearing loss in cognitively normal older adults
Source: Sci Rep. 2019 Aug 29;9:12600. doi: 10.1038/s41598-019-49023-1 (PMC6715732; doi:10.1038/s41598-019-49023-1)
Supplement: Supplementary file 1 — Supplementary Information [file 41598_2019_49023_MOESM1_ESM.pdf]

Supplementary Information

Visual short-term memory binding deficit with hearing loss in cognitively normal older adults

David G. Loughrey, PhD

Mario A. Parra, MD, PhD

Brian A. Lawlor, MD, FRCPI, FRCPsych

Supplementary Table S1: Correlations of AD risk factors with VSTMB task shapes and binding accuracy		
	**Shapes Acc.**	**Bind Acc.**
**AD risk factors**	**TS**	**TS**
**Age**	-0.01 (0.97)	-0.25 (0.11)
**Depression (*CESD-10*)***	0.13 (0.4)	0.23 (0.14)
**Education (*level*)***	0.01 (0.96)	0.03 (0.84)
**Physical inactivity***	0.05 (0.74)	-0.05 (0.74)
**Smoker current/former (*yes/no*)**	0.16 (0.31)	0.11 (0.48)
**Social network (*LSNS*)***	**0.32 (0.04)**	0.14 (0.38)
**Better ear PTA (*WHO*)***	-0.07 (0.68)	**0.35 (0.02)**

TS, total sample; *, Spearman's Rho. Direction of values set so that better function on a measure was positively correlated with better performance on the VSTMB task outcomes. Negative values for age indicated poorer performance with older age. Negative values for current/former smoking indicate poorer performance with non-smoking. CESD-10, Center for Epidemiologic Studies Depression Scale – 10 item; LSNS, Lubben Social Network Scale; PTA, Pure-tone average; VSTMB, visual short-term memory binding; WHO, World Health Organisation.

Supplementary Table S2: Correlations of neuropsychological data with VSTMB task shapes and binding accuracy				
	**Shape Acc.**		**Bind Acc.**	
**NDD tests**	**HLG**	**CG**	**HLG**	**CG**
**FCSRT DFR**	0.21 (0.33)	0.33 (0.18)	0.23 (0.27)	0.08 (0.76)
**Phonemic fluency *(MoCA)***	0.23 (0.27)	-0.37 (0.13)	**0.53 (0.01)**	-0.12 (0.63)
**Semantic fluency *(animals)***	0.04 (0.86)	-0.17 (0.51)	0.17 (0.41)	-0.16 (0.53)
**CRT tot. MRT (*ms*)**	-0.14 (0.51)	-0.19 (0.46)	0.03 (0.88)	0.39 (0.11)
**BNT***	0.06 (0.79)	0.15 (0.55)	*0.36 (0.08)*	-0.02 (0.94)
**MoCA**	-0.06 (0.77)	0.22 (0.39)	0.23 (0.28)	0.23 (0.36)

TS, total sample; *, Spearman's Rho. Direction of values set so that better function on a measure was positively correlated with better performance on the VSTMB task outcomes. BNT, Boston Naming Test 60-item version; CRT, choice-reaction time test with total mean reaction times (MRT) in milliseconds (ms); FCSRT, Free and Cued Selective Reminding Test delayed free recall (DFR) score; MoCA, Montreal cognitive Assessment; NDD, neurodegenerative disease; VSTMB, visual short-term memory binding.

Supplementary Table S3: Correlations of background data with VSTMB task shapes and binding accuracy						
	Shapes Acc.	Bind Acc.	Shapes Acc.		Bind Acc.	
**Demographic**	**TS**	**TS**	**HLG**	**CG**	**HLG**	**CG**
**Age**	-0.01 (0.97)	-0.25 (0.11)	-0.17 (0.42)	0.3 (0.22)	-0.14 (0.51)	-0.27 (0.27)
**Gender***	-0.09 (0.59)	-0.01 (0.93)	-0.01 (0.98)	-0.26 (0.3)	-0.21 (0.3)	0.12 (0.64)
**Education (*years*)**	0.11 (0.47)	-0.02 (0.9)	0.26 (0.21)	-0.24 (0.35)	-0.09 (0.68)	-0.03 (0.92)
**Education *(level)****	0.01 (0.96)	0.03 (0.84)	-0.13 (0.55)	0.24 (0.35)	0.19 (0.37)	-0.27 (0.27)
**Audiological**						
**WHO better ear (*PTA*)***	-0.07 (0.68)	**0.35 (0.02)**	-0.25 (0.23)	0.02 (0.95)	0.08 (0.72)	0.19 (0.46)
**WHO worse ear (*PTA*)***	-0.05 (0.77)	**0.41 (0.01)**	-0.19 (0.37)	0.02 (0.94)	0.11 (0.61)	0.32 (0.2)
**Low freq. better ear (*PTA*)***	-0.08 (0.62)	**0.38 (0.01)**	-0.21 (0.31)	0.08 (0.74)	0.2 (0.35)	-0.04 (0.87)
**Low freq. worse ear (*PTA*)***	-0.10 (0.51)	**0.44 (<0.001)**	-0.34 (0.1)	0.11 (0.65)	0.28 (0.18)	0.08 (0.76)
**High freq. better ear (*PTA*)***	-0.09 (0.57)	**0.31 (0.04)**	-0.2 (0.35)	-0.21 (0.4)	-0.17 (0.43)	**0.51 (0.03)**
**High freq. worse ear (*PTA*)***	-0.05 (0.76)	**0.32 (0.04)**	-0.07 (0.74)	-0.15 (0.55)	-0.15 (0.49)	**0.49 (0.04)**
**Self-rated hearing *(HHIE-S)****	0.04 (0.8)	**0.4 (0.01)**	0.18 (0.39)	0.1 (0.7)	-0.15 (0.49)	-0.34 (0.17)
**Background**						
**Self-rated physical health***	0.2 (0.2)	*0.28 (0.07)*	0.23 (0.27)	0.16 (0.53)	0.12 (0.55)	0.39 (0.11)
**Self-rated mental health***	0.04 (0.78)	0.14 (0.38)	0.12 (0.57)	0.02 (0.95)	0.18 (0.4)	0.13 (0.61)
**Physical inactivity***	0.05 (0.74)	-0.05 (0.74)	0.15 (0.48)	-0.10 (0.70)	0.04 (0.85)	-0.25 (0.32)
**Alcohol consump. *(yes/no)****	*-0.28 (0.07)*	0.09 (0.56)	**-0.44 (0.03)**	-0.07 (0.79)	-0.02 (0.93)	0.28 (0.27)
**Alcohol units *(per wk)****	0.12 (0.52)	0.13 (0.49)	0.11 (0.65)	0.19 (0.52)	0.16 (0.52)	-0.04 (0.9)
**Smoker current *(yes/no)****	0.04 (0.80)	-0.15 (0.33)	0.06 (0.78)	NA	-0.13 (0.54)	NA
**Smoker current/former *(yes/no)****	0.16 (0.31)	0.11 (0.48)	0.1 (0.65)	0.14 (0.58)	0.15 (0.48)	0.22 (0.37)
**Sleep quality *(PSQI)****	**0.33 (0.03)**	-0.05 (0.75)	0.12 (0.56)	**0.62 (0.01)**	-0.18 (0.39)	0.12 (0.63)
**Pre-morbid IQ *(NART)***	-0.04 (0.80)	**0.37 (0.01)**	-0.12 (0.57)	0.11 (0.67)	**0.45 (0.03)**	-0.02 (0.94)
**Self-rated memory***	-0.02 (0.90)	0.06 (0.69)	0.05 (0.82)	0.14 (0.58)	0.16 (0.45)	0.32 (0.2)
**Frailty *(SHARE score)****	-0.08 (0.61)	0.25 (0.11)	0.12 (0.57)	*-0.46 (0.05)*	0.17 (0.43)	0.29 (0.24)
**Depression *(CESD-10)****	0.13 (0.4)	0.23 (0.14)	0.12 (0.56)	0.14 (0.58)	0.2 (0.34)	0.32 (0.2)
**Anxiety *(HADS-A)****	-0.05 (0.74)	0.14 (0.37)	-0.03 (0.89)	-0.07 (0.79)	0.06 (0.78)	0.19 (0.45)
**Apathy *(AES-S)****	**0.41 (0.01)**	0.18 (0.24)	**0.41 (0.04)**	*0.41 (0.09)*	0.12 (0.58)	0.25 (0.33)
**Social network *(LSNS)****	**0.32 (0.04)**	0.14 (0.38)	0.27 (0.19)	0.36 (0.14)	0.1 (0.65)	0.26 (0.29)
**Loneliness *(De Jong Gierveld)****	0.05 (0.75)	0.01 (0.96)	0.03 (0.9)	0.11 (0.67)	-0.16 (0.46)	0.33 (0.18)
**Boredom proneness *(Conroy)****	0.13 (0.41)	-0.03 (0.84)	0.17 (0.43)	0.07 (0.78)	-0.07 (0.74)	0.17 (0.51)
**Perceived stress *(PSS-4)****	-0.19 (0.21)	0.19 (0.22)	0.03 (0.89)	0.4 (0.1)	0.17 (0.42)	0.05 (0.85)

CG, control group; HLG, hearing loss group; TS, total sample; *, Spearman's Rho. Direction of values set so that better function on a measure was positively correlated with better performance on the VSTMB task outcomes. Negative values for age indicated poorer performance with older age. Negative values for gender indicated poorer performance for females. Negative values for alcohol consumption indicate poorer performance with no or fewer units of alcohol consumption. Negative values for current/former smoking indicate poorer performance with non-smoking. AES-S, Apathy Evaluation Scale – Self-rated; CESD-10, Center for Epidemiologic Studies Depression Scale – 10 item; Conroy, Conroy Boredom proneness; DJGLS, 6-item De Jong Gierveld Loneliness Scale; HADS-A, Hospital Anxiety and Depression Scale-Anxiety subscale; HHIE-S, Hearing Handicap Inventory for the Elderly Screening Version; LSNS, Lubben Social Network Scale; NART, National Adult Reading Test; PSQI, Pittsburgh Sleep Quality Index; PSS-4, Perceived Stress Scale-4 item; PTA, Pure-tone average; SHARE, Survey of Health, Ageing and Retirement in Europe Frailty Instrument; VSTMB, visual short-term memory binding; WHO, World Health Organisation.

Supplementary Table S4: Correlations of neuropsychological data with VSTMB task shapes and binding accuracy						
	**Shapes Acc.**	**Bind Acc.**	**Shape Acc.**		**Bind Acc.**	
**Episodic memory**	**TS**	**TS**	**HLG**	**CG**	**HLG**	**CG**
**FCSRT IFR**	0.04 (0.81)	0.22 (0.15)	-0.04 (0.85)	0.27 (0.29)	0.17 (0.42)	0.37 (0.13)
**FCSRT ITR***	-0.1 (0.52)	0.11 (0.49)	-0.14 (0.50)	NA	0.09 (0.66)	NA
**FCSRT DFR**	0.24 (0.13)	0.15 (0.34)	0.21 (0.33)	0.33 (0.18)	0.23 (0.27)	0.08 (0.76)
**FCSRT DTR***	-0.04 (0.8)	*0.26 (0.09)*	-0.06 (0.78)	NA	*0.34 (0.09)*	NA
**WMS-III SSF**	0.22 (0.15)	**0.33 (0.03)**	0.33 (0.11)	0 (>0.99)	0.33 (0.11)	**0.49 (0.04)**
Comp. z-score	0.22 (0.15)	**0.32 (0.04)**	0.21 (0.32)	0.27 (0.28)	0.31 (0.13)	*0.45 (0.06)*
**Executive function**						
**CAMDEX VR**	0.19 (0.22)	**0.39 (0.01)**	**0.4 (0.048)**	-0.16 (0.52)	**0.49 (0.01)**	0.23 (0.36)
**SART com. errors***	-0.25 (0.1)	0.09 (0.57)	-0.29 (0.17)	-0.18 (0.48)	0.15 (0.48)	0.14 (0.58)
**SART om. errors***	-0.07 (0.64)	0.17 (0.27)	-0.12 (0.58)	-0.02 (0.94)	0.07 (0.73)	**0.57 (0.01)**
**SART total errors***	-0.12 (0.45)	0.17 (0.27)	-0.18 (0.40)	-0.09 (0.71)	0.11 (0.60)	**0.55 (0.02)**
**Phon. fluency *(MoCA)***	0.04 (0.82)	*0.3 (0.05)*	0.23 (0.27)	-0.37 (0.13)	**0.53 (0.01)**	-0.12 (0.63)
**WMS-III SSB**	0.14 (0.36)	**0.36 (0.02)**	0.22 (0.29)	0 (>0.99)	*0.36 (0.08)*	*0.43 (0.08)*
**WMS-III SST**	0.21 (0.18)	**0.39 (0.01)**	0.32 (0.12)	0 (>0.99)	*0.39 (0.05)*	**0.52 (0.03)**
Comp. z-score	0.011(0.94)	**0.39 (0.01)**	0.18 (0.38)	-0.25 (0.32)	**0.52 (0.01)**	*0.43 (0.08)*
**Processing speed**						
**CRT mot. MRT (*ms*)**	*-0.26 (0.1)*	0.07 (0.65)	-0.31 (0.13)	-0.12 (0.63)	0.03 (0.87)	0.16 (0.54)
**CRT cog. MRT (*ms*)***	0.02 (0.89)	0.09 (0.59)	0.25 (0.22)	-0.18 (0.47)	-0.02 (0.93)	0.33 (0.18)
**CRT tot. MRT (*ms*)**	0.16 (0.32)	-0.1 (0.52)	-0.14 (0.51)	-0.19 (0.46)	0.03 (0.88)	0.39 (0.11)
**SART tot. MRT *(ms)***	0.24 (0.13)	0.13 (0.42)	0.06 (0.76)	0.17 (0.49)	-0.15 (0.47)	*0.43 (0.08)*
**Language**						
**BNT***	0.11 (0.50)	0.24 (0.13)	0.06 (0.79)	0.15 (0.55)	*0.36 (0.08)*	-0.02 (0.94)
**Sem. fluency *(animals)***	-0.04 (0.8)	0.06 (0.69)	0.04 (0.86)	-0.17 (0.51)	0.17 (0.41)	-0.16 (0.53)
Comp. z-score	0.08 (0.61)	*0.28 (0.07)*	0.11 (0.59)	-0.01 (0.96)	*0.4 (0.05)*	-0.16 (0.52)
**Visuospatial ability**						
**MCG complex figure**	0.05 (0.77)	**0.43 (0.004)**	-0.02 (0.94)	0.12 (0.63)	**0.47 (0.02)**	0.16 (0.53)
**Global cognition**						
**MoCA**	0.03 (0.84)	0.21 (0.18)	-0.06 (0.77)	0.22 (0.39)	0.23 (0.28)	0.23 (0.36)
**MoCA adj.#**	0.18 (0.25)	*0.29 (0.06)*	0.21 (0.32)	0.15 (0.56)	**0.41 (0.04)**	0.15 (0.57)
Comp. global z-score+	0.05 (0.76)	**0.51 (0.001)**	0.09 (0.67)	-0.07 (0.8)	**0.52 (0.01)**	**0.48 (0.04)**
**Bind Acc.**	0.13 (0.39)	.	0.12 (0.57)	0.14 (0.58)	.	.

CG, control group; HLG, hearing loss group; TS, total sample; *, Spearman's Rho; #, MoCA score with audiological items removed.
+, Composite global z-score calculated from the mean of the composite scores for episodic memory (except FCSRT total scores), executive functions (except SART and WMS-III SS total scores) and language, and from processing speed (CRT total MRT), and visuospatial ability. Direction of values set so that better function on a measure was positively correlated with better performance on the VSTMB task outcomes. Correlations not available for FCSRT immediate and delayed total scores in CG as all participants obtained a perfect score of 16. BNT, Boston Naming Test 60-item version; CAMDEX VR, Cambridge Mental Disorders of the Elderly Examination battery Visual Reasoning subtest; CRT, choice-reaction time test which included motor, cognitive and total mean reaction times (MRT); FCSRT, Free and Cued Selective Reminding Test including immediate (IFR) and delayed free recall (DFR) scores and immediate (ITR) and delayed (DTR) total recall scores; MCG, Medical College of Georgia Complex Figure test copy; MoCA, Montreal cognitive Assessment; SART, Sustained Attention to Response task including commission, omission total error scores and mean reaction time (MRT); VSTMB, visual short-term memory binding; WMS-III, Wechsler Memory Scale-III (SSF) spatial span forward, (SSB) backward and (SST) total scores.

Supplementary Table S5: Group differences on background data between high and low VSTMB task binding accuracy performers										
			HLG-low vs. HLG-high vs. CG			HLG-low vs. HLG-high vs. CG-high			HLG-low vs. HLG-high	
	HLG-low	HLG-high	CG	Significance test		CG-high	Significance test		Significance test	
	M (SD)	*M (SD)*	*M (SD)*	*F*	*p*	*M (SD)*	*F*	*p*	*F*	*p*
**Demographic**										
**N**	14	11	18			14				
**Age**	72.93 (5.7)	72.09 (6.14)	69.11 (6.63)	1.67	0.20	68.36 (6.42)	2.20	0.13	0.35	0.73
**Gender *(female/male)***	8/6	6/5	14/4	-	0.34^{+}	11/3	-	0.45^{+}	0.02	0.90
**Education *(years)***	14.32 (3.62)	12.64 (3.56)	14.44 (3.09)	1.10	0.34	14.5 (2.41)	1.21	0.31	1.16	0.26
**Education *(level)***	2.79 (0.89)	2.82 (0.6)	2.94 (0.73)	0.58*	0.75	2.86 (0.77)	0.08*	0.78	76.5*	0.98
**Audiological**										
**WHO better ear PTA**	52.41 (16.71)	46.7 (18.15)	13.61 (6.61)	31.02*	**<0.01**	12.68 (6.02)	20.32*	**<0.01**	60.00*	0.35
**WHO worse ear PTA**	68.66 (26.65)	56.25 (23.98)	19.17 (8.73)	29.95*	**<0.01**	17.23 (8.04)	20.34*	**<0.01**	55.50*	0.24
**Low freq. better ear PTA**	45.95 (17.56)	31.36 (22.92)	7.96 (4.9)	24.90*	**<0.01**	8.1 (5.1)	20.40*	**<0.01**	45.50*	*0.08*
**Low freq. worse ear PTA**	60.36 (24.67)	41.67 (30.05)	13.24 (7.06)	24.69*	**<0.01**	12.86 (7.26)	19.52*	**<0.01**	42.00*	*0.06*
**High freq. better ear PTA**	64.76 (18.44)	64.7 (16.81)	23.7 (10.79)	29.90*	**<0.01**	20.95 (9.76)	19.90*	**<0.01**	76.50*	0.98
**High freq. worse ear PTA**	79.4 (27.46)	74.09 (18.35)	32.13 (13.4)	27.67*	**<0.01**	28.45 (12.22)	18.48*	**<0.01**	72.00*	0.78
**Self-rated hearing *(HHIE-S)***	20.29 (7.64)	19.64 (9.71)	4.44 (6.49)	21.22*	**<0.01**	3.43 (4.03)	21.35*	**<0.01**	74.50*	0.89
**Background**										
**Self-rated physical health**	3.57 (0.94)	3.45 (1.13)	3.83 (1.04)	0.94*	0.63	4.07 (0.83)	1.84*	0.18	73.00*	0.82
**Self-rated mental health**	3.93 (1)	4 (0.63)	3.94 (1.11)	0.02*	0.99	4.07 (1.07)	0.19*	0.66	76.00*	0.95
**Physical inactivity**	2 (1.04)	2.09 (1.04)	2 (0.77)	0.06*	0.97	2.14 (0.77)	0.28*	0.87	-0.22	0.83
**Alcohol consumption *(yes/no)***	11/3	8/3	14/4	-	>0.99^{+}	10/4	-	>0.99^{+}	-	>0.99^{+}
**Alcohol units *(per wk)***	8.02 (6.77)	9.13 (7.99)	12.64 (11.74)	1.34*	0.51	13.4 (13.47)	0.98*	0.32	41.50*	0.84
**Smoker current *(yes/no)***	1/13	0/11	0/18	-	0.58^{+}	0/14	-	>0.99^{+}	-	>0.99^{+}
**Smoker former *(yes/no)***	5/9	5/6	7/11	-	0.93^{+}	6/8	-	0.92^{+}	-	0.7^{+}
**Sleep quality *(PSQI)***	4.64 (3.03)	6 (2.97)	4.78 (2.53)	1.80*	0.41	4.07 (1.82)	0.11*	0.75	55.50*	0.24
**Pre-morbid IQ *(NART)***	110.88 (6.25)	115.39 (6.48)	115.17 (5.38)	2.55	*0.09*	115.02 (5.17)	2.35	0.11	-1.76	*0.09*
**Self-rated memory**	3.36 (0.84)	3.27 (0.9)	3.56 (0.86)	0.97*	0.62	3.57 (0.94)	0.47*	0.50	72.00*	0.77
**Frailty *(SHARE score)***	0.24 (0.85)	0.28 (0.95)	0.21 (1.07)	0.17*	0.92	-0.06 (0.55)	0.72*	0.40	74.00*	0.87
**Depression *(CESD-10)***	4.86 (3.18)	3.45 (2.73)	4.83 (4.46)	1.28*	0.53	3.5 (2.59)	1.40*	0.24	57.50*	0.28
**Anxiety *(HADS-A)***	3.71 (2.81)	3.18 (2.36)	3.83 (3.5)	0.09*	0.96	3 (2.83)	0.66*	0.42	71.50*	0.76
**Apathy *(AES-S)***	26.86 (3.74)	27 (5.8)	27.83 (7.21)	0.21*	0.90	26.21 (6.65)	1.28*	0.26	67.50*	0.60
**Social network *(LSNS)***	19.93 (5.11)	21.36 (6.23)	19.5 (6.17)	0.45*	0.80	20.71 (5.15)	0.42*	0.52	65.00*	0.51
**Loneliness *(De Jong Gierveld)***	0.14 (0.36)	0.55 (0.93)	0.83 (1.62)	2.20*	0.33	0.36 (0.84)	0.31*	0.58	59.00*	0.19
**Boredom proneness *(Conroy)***	1.29 (0.47)	1.45 (0.69)	1.61 (0.7)	1.86*	0.39	1.43 (0.51)	0.60*	0.44	69.00*	0.59
**Perceived stress *(PSS-4)***	3.29 (2.27)	2.82 (2.14)	2.33 (2.72)	2.42*	0.30	1.71 (2.16)	3.92*	0.05	66.50*	0.56

CG, control group; CG-high, control group high performers; HLG-high, hearing loss group high performers; HLG-low, hearing loss group low performers; *, Kruskal-Wallis test/Mann-Whitney U; $^{+}$, Fisher's exact test.

Supplementary Table S6: Group differences on neuropsychological data between high and low VSTMB task binding accuracy performers

			HLG-low vs. HLG-high vs. CG			HLG-low vs. HLG-high vs. CG-high			HLG-low vs. HLG-high	
	**HLG-low**	**HLG-high**	**CG**	**Significance test**		**CG-high**	**Significance test**		**Significance test**	
	M (SD)	*M (SD)*	*M (SD)*	*F*	*p*	*M (SD)*	*F*	*p*	*F*	*p*
**Episodic memory**										
**FCSRT IFR**	32.93 (7.48)	33.73 (7.14)	34.39 (4.35)	0.22	0.81	35.14 (4)	0.44	0.65	-0.27	0.79
**FCSRT ITR**	47.43 (1.87)	47.91 (0.3)	48 (0)	2.55*	0.28	48 (0)	2.04*	0.36	-0.84	0.41
**FCSRT DFR**	12.43 (2.95)	12.64 (2.58)	12.11 (2.06)	0.16	0.85	12.07 (2.13)	0.16	0.86	-0.18	0.86
**FCSRT DTR**	15.86 (0.54)	16 (0)	16 (0)	2.07*	0.36	16 (0)	1.79*	0.41	-0.88	0.39
**WMS-III SSF**	6.57 (1.91)	7.73 (2.1)	7 (1.82)	1.12	0.34	7.36 (1.87)	1.17	0.32	-1.44	0.16
Comp. z-score	-0.12 (0.83)	0.16 (0.89)	-0.01 (0.58)	0.40	0.67	0.06 (0.57)	0.45	0.64	-0.82	0.42
**Executive function**										
**CAMDEX VR**	3.36 (1.22)	4.09 (0.94)	3.83 (1.25)	1.30	0.28	4.07 (1.27)	1.72	0.19	-1.65	0.11
**SART com. errors**	3.21 (2.86)	3 (1.67)	3.89 (2.97)	0.55*	0.76	3.86 (2.98)	0.45*	0.80	76*	0.96
**SART om. errors**	6.79 (6.72)	5.55 (3.98)	10.33 (10.34)	1.35*	0.51	7.86 (8.66)	0.13*	0.94	74.5*	0.89
**SART total errors**	10 (9.02)	8.55 (4.95)	14.22 (11.56)	1.84*	0.40	11.71 (9.9)	0.47*	0.79	77*	>0.99
**Phon. fluency**	13.29 (4.2)	17.27 (4.74)	14.22 (4.17)	2.81	*0.07*	14.21 (4.44)	2.64	*0.09*	-2.23	**0.04**
**WMS-III SSB**	6 (1.3)	7 (2.15)	6.67 (1.82)	1.08	0.35	7 (1.84)	1.44	0.25	-1.44	0.16
**WMS-III SST**	12.57 (2.41)	14.73 (4.03)	13.67 (3.2)	1.40	0.26	14.36 (3.25)	1.68	0.20	-1.57^{+}	0.14
Comp. z-score	-0.14 (0.59)	0.32 (0.50)	-0.08 (0.7)	2.00	0.15	-0.01 (0.71)	1.69	0.20	3.76	*0.07*
**Processing speed**										
**CRT mot. MRT (ms)**	303.17 (75.87)	302.71 (81.35)	297.3 (56.19)	0.04	0.97	291.89 (49.57)	0.12	0.89	-0.12	0.91
**CRT cog. MRT (ms)**	488.99 (71.6)	481.21 (62.01)	501.85 (66.15)	1.02*	0.60	489.09 (67.61)	0.25*	0.88	68*	0.62
**CRT tot. MRT (ms)**	792.57 (95.09)	783.24 (74.58)	797.43 (84.53)	0.09	0.91	779.07 (76.34)	0.10	0.91	-0.23	0.82
**SART tot. MRT (ms)**	338.19 (75.68)	328.95 (93.56)	319.14 (62.53)	0.25	0.78	310.72 (63.64)	0.46	0.64	-0.62	0.54
**Language**										
**BNT**	54.93 (3.87)	56.55 (3.17)	56.5 (2.33)	1.51*	0.47	56.5 (2.38)	1.48*	0.48	58*	0.29
**Sem. fluency**	22 (3.23)	23.91 (7.45)	22.83 (6.36)	0.33	0.72	22.79 (6.9)	0.31	0.74	-0.79^{+}	0.44
Comp. z-score	-0.24 (0.75)	0.18 (1.00)	0.08 (0.69)	1.02	0.37	0.08 (0.73)	0.93	0.40	1.44	0.24
**Visuospatial ability**										
**MCG complex figure**	23.5 (4.26)	25.14 (4.57)	27.06 (4.5)	2.55	*0.09*	27.43 (4.01)	3.00	*0.06*	-0.92	0.37
**Global cognition**										
**MoCA**	25.86 (2.91)	26.09 (2.91)	26 (2.74)	0.02	0.98	26.36 (2.87)	0.10	0.90	-0.20	0.84
**MoCA adj.***	17.57 (1.79)	17.91 (1.87)	17.72 (2.02)	0.10	0.91	17.93 (2.02)	0.15	0.86	-0.46	0.65
Global z-score+	-0.18 (0.46)	0.14 (0.57)	0.06 (0.42)	1.68	0.20	0.13 (0.37)	2.37	0.11	2.54	0.13
**VSTMB outcomes**										
**Shape mean RT (ms)**	2141.14 (296.82)	2170.27 (568.69)	2061.61 (319.21)	0.27*	0.87	2004.36 (289.74)	0.76*	0.68	67*	0.58
**Shape A’**	0.95 (0.06)	0.95 (0.03)	0.98 (0.03)	1.08*	0.58	0.99 (0.03)	2.95*	0.23	62.5*	0.40
**Shape Acc.**	0.96 (0.07)	0.98 (0.02)	0.96 (0.04)	0.55*	0.76	0.96 (0.04)	1.05*	0.59	74.5*	0.88
**Bind mean RT (ms)**	2696.86 (595.62)	2391.18 (455.34)	2330.11 (559.68)	3.57*	0.17	2181.14 (491.34)	6.41*	**0.04**	49*	0.13
**Bind A’**	0.79 (0.09)	0.96 (0.03)	0.92 (0.08)	20.74*	**<0.001**	0.95 (0.04)	24.11*	**<0.001**	0*	**<0.001**
**Bind Acc.**	0.67 (0.24)	0.96 (0.04)	0.93 (0.06)	24.12*	**<0.001**	0.96 (0.03)	26.98*	**<0.001**	4*	**<0.001**

CG, control group; CG-high, control group high performers; HLG-high, hearing loss group high performers; HLG-low, hearing loss group low performers; *, Kruskal-Wallis test/Mann-Whitney U.

Supplementary References

S1. Buysse DJ, Reynolds CF, 3rd, Monk TH, Berman SR, Kupfer DJ. The Pittsburgh Sleep Quality Index: a new instrument for psychiatric practice and research. Psychiatry research 1989;28:193-213.

S2. Nelson HE. National Adult Reading Test (NART): For the Assessment of Premorbid Intelligence in Patients with Dementia: Test Manual: NFER-Nelson, 1982.

S3. Romero-Ortuno R, Walsh CD, Lawlor BA, Kenny RA. A frailty instrument for primary care: findings from the Survey of Health, Ageing and Retirement in Europe (SHARE). BMC geriatrics 2010;10:57.

S4. Radloff LS. The CES-D Scale. Applied Psychological Measurement 1977;1:385-401.

S5. Zigmond AS, Snaith RP. The hospital anxiety and depression scale. Acta psychiatrica Scandinavica 1983;67:361-370.

S6. Marin RS, Biedrzycki RC, Firinciogullari S. Reliability and validity of the Apathy Evaluation Scale. Psychiatry research 1991;38:143-162.

S7. Lubben J, Blozik E, Gillmann G, et al. Performance of an abbreviated version of the Lubben Social Network Scale among three European community-dwelling older adult populations. The Gerontologist 2006;46:503-513.

S8. De Jong Gierveld J, Van Tilburg T. A 6-item scale for overall, emotional, and social loneliness confirmatory tests on survey data. Research on Aging 2006;28:582-598.

S9. Conroy RM, Golden J, Jeffares I, O'Neill D, McGee H. Boredom-proneness, loneliness, social engagement and depression and their association with cognitive function in older people: a population study. Psychol Health Med 2010;15:463-473.

S10. Cohen S, Kamarck T, Mermelstein R. A global measure of perceived stress. Journal of health and social behavior 1983;24:385-396.

S11. Ventry IM, Weinstein BE. Identification of elderly people with hearing problems. Asha 1983;25:37-42.

S12. Nasreddine ZS, Phillips NA, Bedirian V, et al. The Montreal Cognitive Assessment, MoCA: a brief screening tool for mild cognitive impairment. Journal of the American Geriatrics Society 2005;53:695-699.

S13. Grober E, Buschke H, Crystal H, Bang S, Dresner R. Screening for dementia by memory testing. Neurology 1988;38:900-903.

S14. Wechsler D. Wechsler Memory Scale—Third edition. Administration and scoring manual. USA: The Psychological Corporation, 1997.

S15. Roth M, Tym E, Mountjoy CQ, et al. CAMDEX. A standardised instrument for the diagnosis of mental disorder in the elderly with special reference to the early detection of dementia. The British journal of psychiatry : the journal of mental science 1986;149:698-709.

S16. Robertson IH, Manly T, Andrade J, Baddeley BT, Yiend J. 'Oops!': performance correlates of everyday attentional failures in traumatic brain injured and normal subjects. Neuropsychologia 1997;35:747-758.

S17. Donoghue O, Feeney J, O'Leary N, Kenny RA. Baseline Mobility is Not Associated with Decline in Cognitive Function in Healthy Community-Dwelling Older Adults: Findings From The Irish Longitudinal Study on Ageing (TILDA). The American journal of geriatric psychiatry : official journal of the American Association for Geriatric Psychiatry 2018;26:438-448.

S18. Kaplan E, Goodglass H, Weintraub S. Boston Naming Test (2nd ed.). Philadelphia: Lippincott, Williams, & Wilkins, 2001.

S19. Lezak, MD. Neuropsychological Assessment. Oxford University Press, 2004.

S20. Loring DW, Meador KJ. The Medical College of Georgia (MCG) Complex Figures: Four forms for follow-up. In: A. KJ, E. K, eds. The handbook of Rey-Osterrieth Complex Figure: Clinical and research applications. Lutz, FL: Psychological Assessment Resources, 2003: 313–321.

S21. Dupuis K, Pichora-Fuller MK, Chasteen AL, Marchuk V, Singh G, Smith SL. Effects of hearing and vision impairments on the Montreal Cognitive Assessment. Aging, Neuropsychology, and Cognition 2015;22:413-437.
